# PI3K/AKT signaling activates HIF1α to modulate the biological effects of invasive breast cancer with microcalcification

**DOI:** 10.1038/s41523-023-00598-z

**Published:** 2023-11-13

**Authors:** Yao Tian, Lu Zhao, Zhengwei Gui, Shiyang Liu, Chenguang Liu, Tianyao Yu, Lin Zhang

**Affiliations:** Department of Thyroid and Breast Surgery, Tongji Hospital of Tongji Medical College of Huazhong University of Science and Technology, 1095 Jiefang Avenue, Qiaokou District, Wuhan, Hubei Province 430030 China

**Keywords:** Breast cancer, Cancer microenvironment

## Abstract

Microcalcification (MC) is a valuable diagnostic indicator of breast cancer, and it is reported to be associated with increased tumor aggressiveness and poor prognosis. Nevertheless, the exact potential molecular mechanism is not completely understood. Here, we find that the mineralized invasive breast cancer (IBC) cells not only increased their proliferation and migration, but also showed the characteristic of doxorubicin resistance. The PI3K/AKT signaling pathway is associated with the generation of calcification in IBC, and it activates the transcription and translation of its downstream hypoxia-inducible factor 1α (HIF1α). Knockdown of HIF1α protein significantly downregulated cell proliferation and migration while calcification persists. Meanwhile, calcified breast cancer cells restored sensitivity to doxorubicin because of suppressed HIF1α expression. In addition, we provide initial data on the underlying value of HIF1α as a biomarker of doxorubicin resistance. These findings provide a new direction for exploring microcalcifications in IBC.

## Introduction

In recent years, the global incidence of breast cancer continues to rise, surpassing lung cancer with an 11.7% incidence in 2020, becoming the most common tumor in the world, and will continue to have a significant effect on cancer deaths worldwide^1^. By 2040, only due to population growth and aging, the burden of breast tumor is projected to increase to over three million new cases and one million deaths annually^2^. Although the treatments of breast cancer have made great progress, the five-year survival rate of patients with early breast cancer can reach more than 85% and >120,000 people are expected to die from breast cancer in China by 2022^3,4^. Therefore, the development of accurate molecular diagnosis and prognosis is crucial for individualized treatment and precise treatment of breast cancer patients.

Breast tumor can be detected by X-ray mammography. Sometimes, microcalcification (MC) on radiographic imaging may be the only marker of breast tumors^5,6^. In addition to being used as a detection marker, MC in breast cancer may also be related to prognostic value. There are several studies that highlighted the link between calcification and poor prognosis^7–9^. Studies have also shown that MC in invasive breast cancer (IBC) intensifies the epithelial-mesenchymal transition of breast tumor cells and promotes the occurrence of bone metastasis^10,11^. Furthermore, several researchers have reported the association between the development and metastasis of IBC and molecules related to MC, including BMP2, RUNX2, OPN, OCN, and ALP^12–15^. These molecules may result in poor prognosis and should be valued. Therefore, it is vital to determine the molecular mechanism of MC formation and to evaluate its clinical importance on the development of IBC.

Under hypoxic conditions, hypoxia-inducible factor 1 α (HIF1α) is a major regulator that promotes tumor development and increases glycolysis. The expression of HIF1α increases glucose flux and enhance glycolytic by upregulating glycolytic enzymes and membrane transporters^16,17^. A study shows that the calcification and osteogenic transformation of vascular smooth muscle cells is promoted by HIF1α/pyruvate dehydrogenase kinase 4 (PDK4), which is activated by advanced glycosylation products^18^. However, HIF1α is also reported to be the major adjuster of tumor progression and recurrence under normoxia^19^. It is important to note that the role of HIF1α in glucose metabolism and chemotherapy resistance in IBC MC remains to be fully explored. Furthermore, elucidating the mechanism of stabilization and activation of HIF1α under normoxia is of great significance.

Metabolism is one of the important markers of tumor transformation, especially metabolic reprogramming^20,21^. Metabolic reprogramming usually implies a reduction of the mitochondrial oxidative phosphorylation (OXPHOS) system in cancer cells. Even oxygen is abundant, the energy is supplied mainly through glycolysis or pentose phosphate pathways (PPP). Meanwhile, the major site of energy production transfers from mitochondria to cytoplasm^22,23^. The cell metabolic transformation is also a key participant to tumor development and chemoresistance except for ensuring energy supply^24,25^. A recent study has highlighted that there is an enhanced mitochondrial OXPHOS in the calcified cancer cell, which is associated with epithelial interstitial transformation^10^.

In the research, we attempt to illustrate the effect of MC on IBC and its mechanism of formation by establishing a rapidly stable calcification model of breast cancer cells. We observed that HIF1α, as a downstream molecule of PI3K/AKT signaling, is related to very important biological processes and even in doxorubicin resistance in IBC with calcification.

## Results

### Establishment of human breast cancer cell calcification model in vitro

**Fig. 1:**
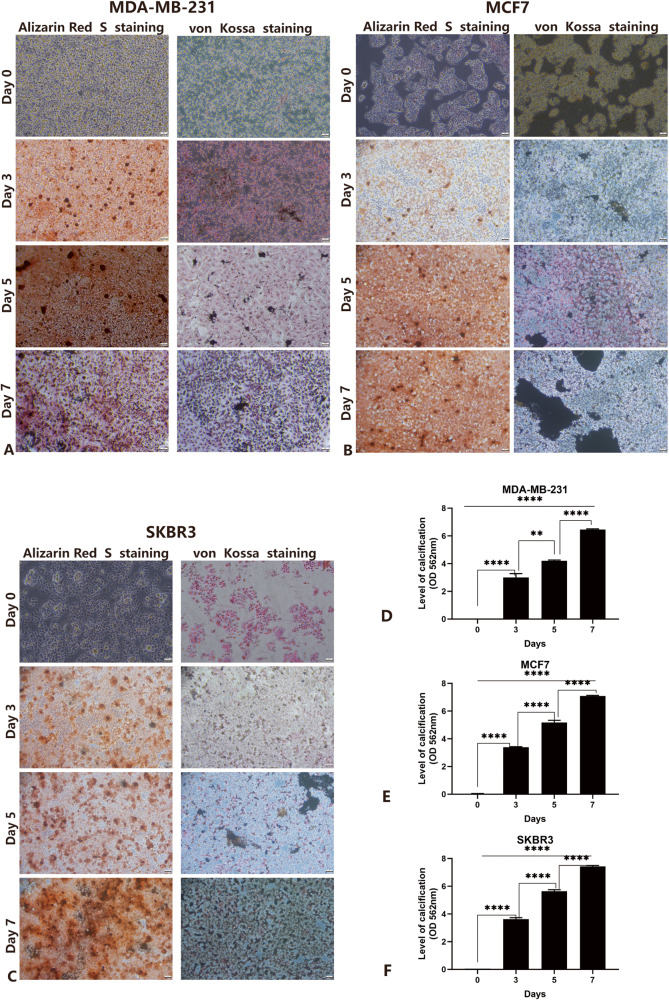
The qualitative and quantitative analysis of calcification at different time points. Scale bars =50 µm. **A**–**C** Alizarin Red S staining and von Kossa staining in MDA-MB-231 cell lines (**A**), MCF7 cell lines (**B**), and SKBR3 cell lines (**C**); **D**–**F**. The quantification of calcification in MDA-MB-231 cell lines (**D**), MCF7 cell lines (**E**), and SKBR3 cell lines (**F**). Data were analyzed by ANOVA and chi-square test. Remarks: ns *p* ≥ 0.05, *p < 0.05, **p < 0.01, ***p < 0.001, ****p < 0.0001; Data are presented as mean ± SD. Bar: mean value; error bar: standard error.

**Fig. 2:**
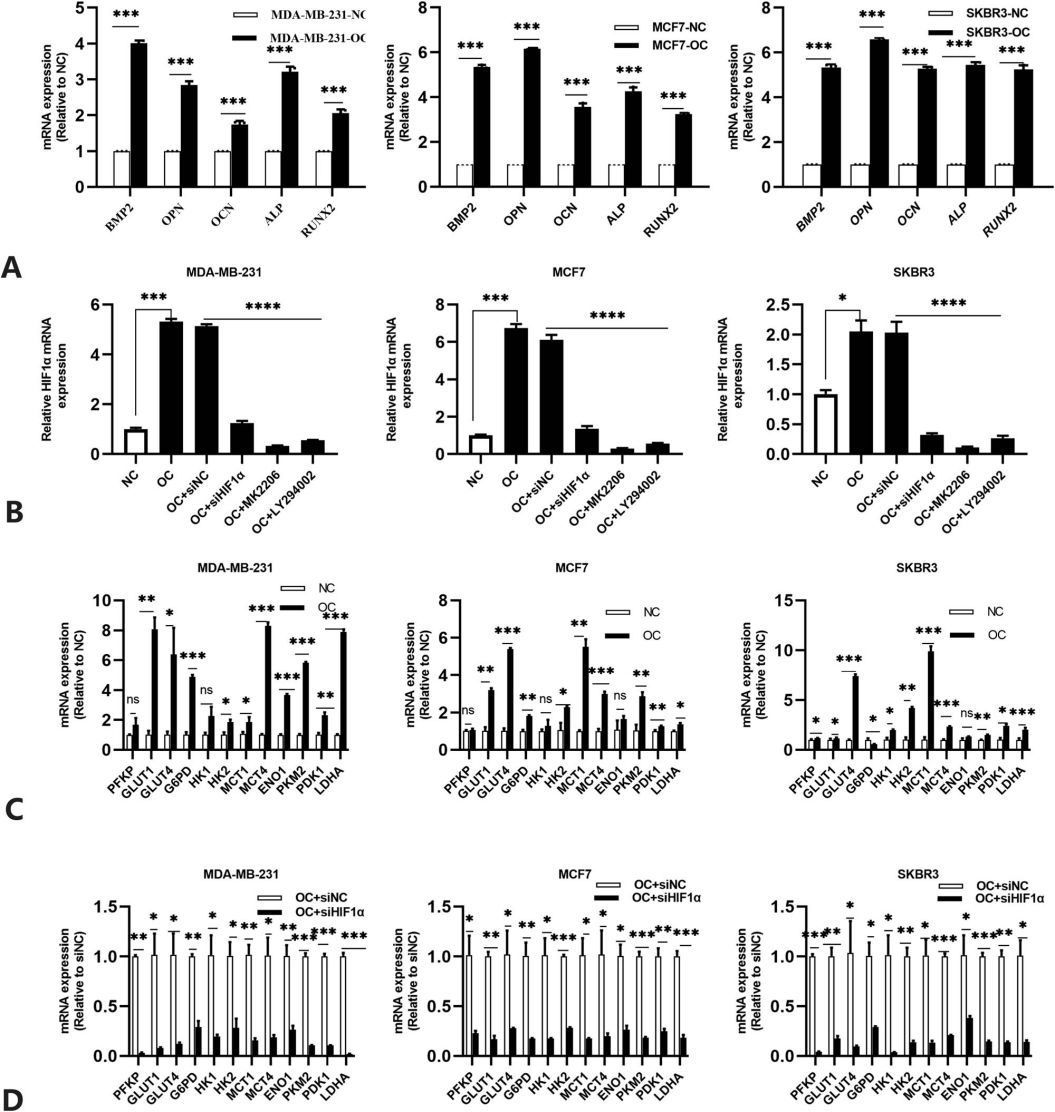
mRNA expressions of the different molecules in MDA-MB-231, MCF7, and SKBR3 cells were detected through quantitative real-time PCR and expressed as a ratio to GAPDH. **A** Calcification-related biomarkers; **B** mRNA expression levels of HIF1α under different conditions; **C** mRNA levels of the glycolytic enzymes and membrane transporters in OC-treated cells compared to normal cells; **D** mRNA levels of the glycolytic enzymes and membrane transporters in OC-treated cells with HIF1α knockdown. Data were analyzed by ANOVA and Student’s *t* test. Remarks: ns *p* ≥ 0.05, *p < 0.05, **p < 0.01, ***p < 0.001, ****p < 0.0001; data are presented as mean ± SD. Bar: mean value; error bar: standard error.

To explore the molecular mechanisms of MC in IBC, we developed a model that mimics MC of breast tumor cells in vitro. Human breast cancer cells MDA-MB-231 (triple-negative), SKBR3 (HER2 overexpression), and MCF7 (luminal-A type) were cultured in DMEM medium containing Osteogenic Cocktail. Significant red calcified nodules were detected after 48 hours, and black calcified nodules were visible through von Kossa staining. The results showed that staining intensity increased over time (Fig. 1A–C). In addition, we used 10% cetylpyridinium chloride to extract alizarin red S and determined the concentration of dye at the OD of 562 nm to quantify the calcified nodules. Apparently, we obtained results consistent with the staining results (Fig. 1D–F). Interestingly, MCF7 cells were similar to SKBR3 cells, both more sensitive to osteogenic differentiation and easier and faster to produce calcified nodules than MDA-MB-231 cells. Quantitative real-time PCR (qRT-PCR) confirmed an increase in calcification-related biomarkers (BMP2, OPN, OCN, ALP, RUNX2) in IBC cell lines cultured with calcified medium (Fig. 2A). Together, these data suggested that OC can promote calcification of IBC cells in vitro and can produce significant mineralized nodules only 48 hours. For the subsequent mechanism studies, it is an efficient and fast calcification model in vitro.

### Enhanced proliferation, invasion, migration, and activation of PI3K/AKT signaling in IBC cells under OC induction

**Fig. 3:**
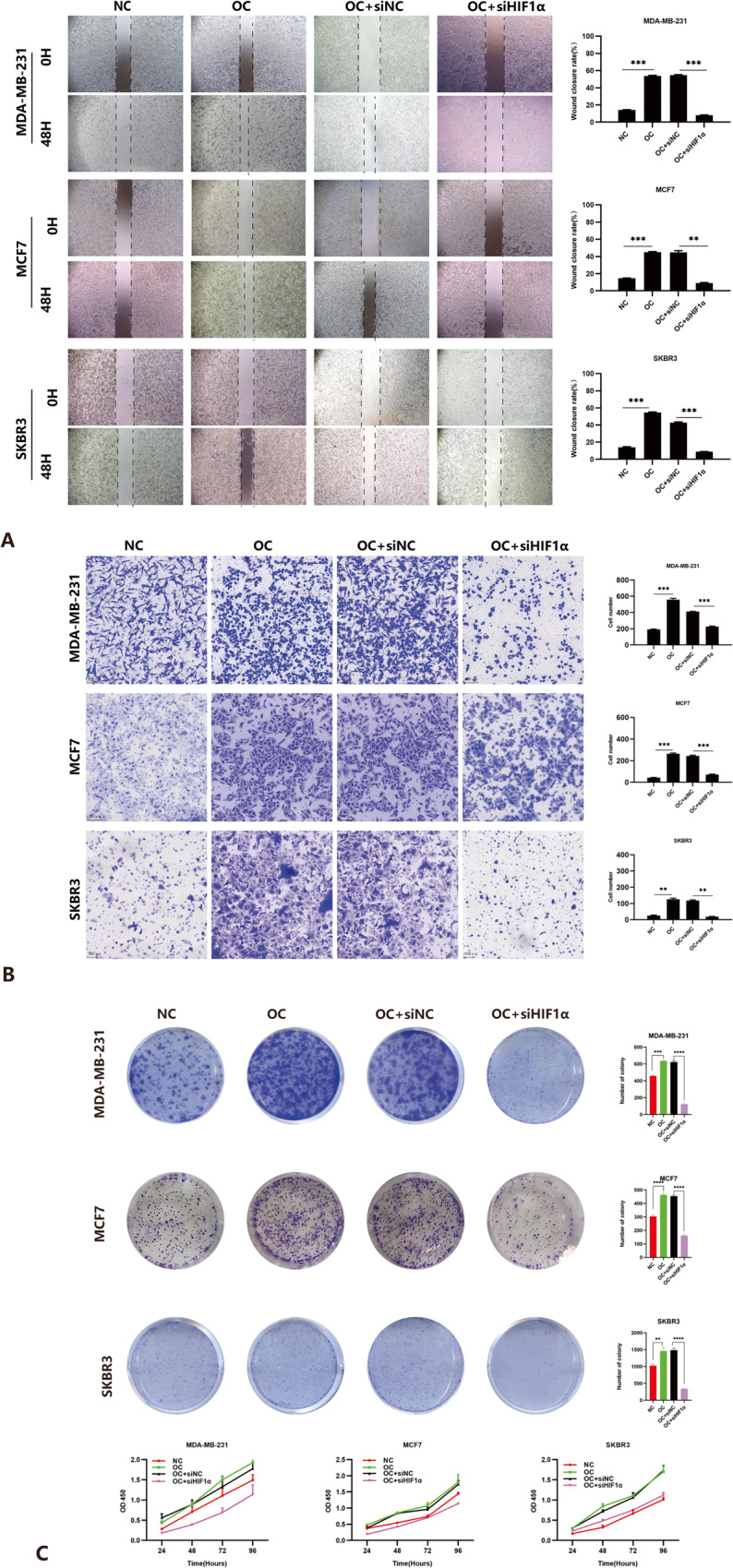
HIF1α promotes the proliferation, migration and invasion of calcified breast cancer cells. **A** Cell migration was obviously increased when cultured with osteogenic cocktail (OC) while the migration was significantly inhibited when Hypoxia-inducible factor 1α (HIF1α) was knocked down in the wound-healing assay. **B** Enchanced migration of calcified cells was inhibited when HIF1α was knocked down in the transwell assay, Scale bars =100 µm. **C** Colony formation assay (left) and CCK8 assay (right) both showed increased proliferation in calcified cancer cells and knockdown of HIF1α inhibited the proliferation. Data were analyzed by ANOVA and Student’s *t* test. Remarks: **p < 0.01, ***p < 0.001, ****p < 0.0001; Data are presented as mean ± SD. Bar: mean value; error bar: standard error.

In the research, the experimental group was breast cancer cells treated with OC for 48 hours, and the control group was cultured with normal medium for the same time. We utilized a series of cell function experiments to explore the migration, invasion, and proliferation ability of calcified IBC cells. Migration of breast cancer cells grown in OC medium was significantly increased (Fig. 3A). In addition, calcified breast cancer cells also had a stronger invasion ability. Certainly, the MDA-MB-231 breast cells were more aggressive with or without MC than other two cell lines (Fig. 3B). CCK8 proliferation and clone formation experiments showed that the proliferation ability of OC-treated IBC cells was also significantly enhanced compared to control groups (Fig. 3C). In the course of the experiment, we found that calcified breast cancer cells were better at sticking to walls. As was shown in Fig. 3, compared to the migration of calcified breast cancer cells, there were strong effects of cell proliferation.

**Fig. 4:**
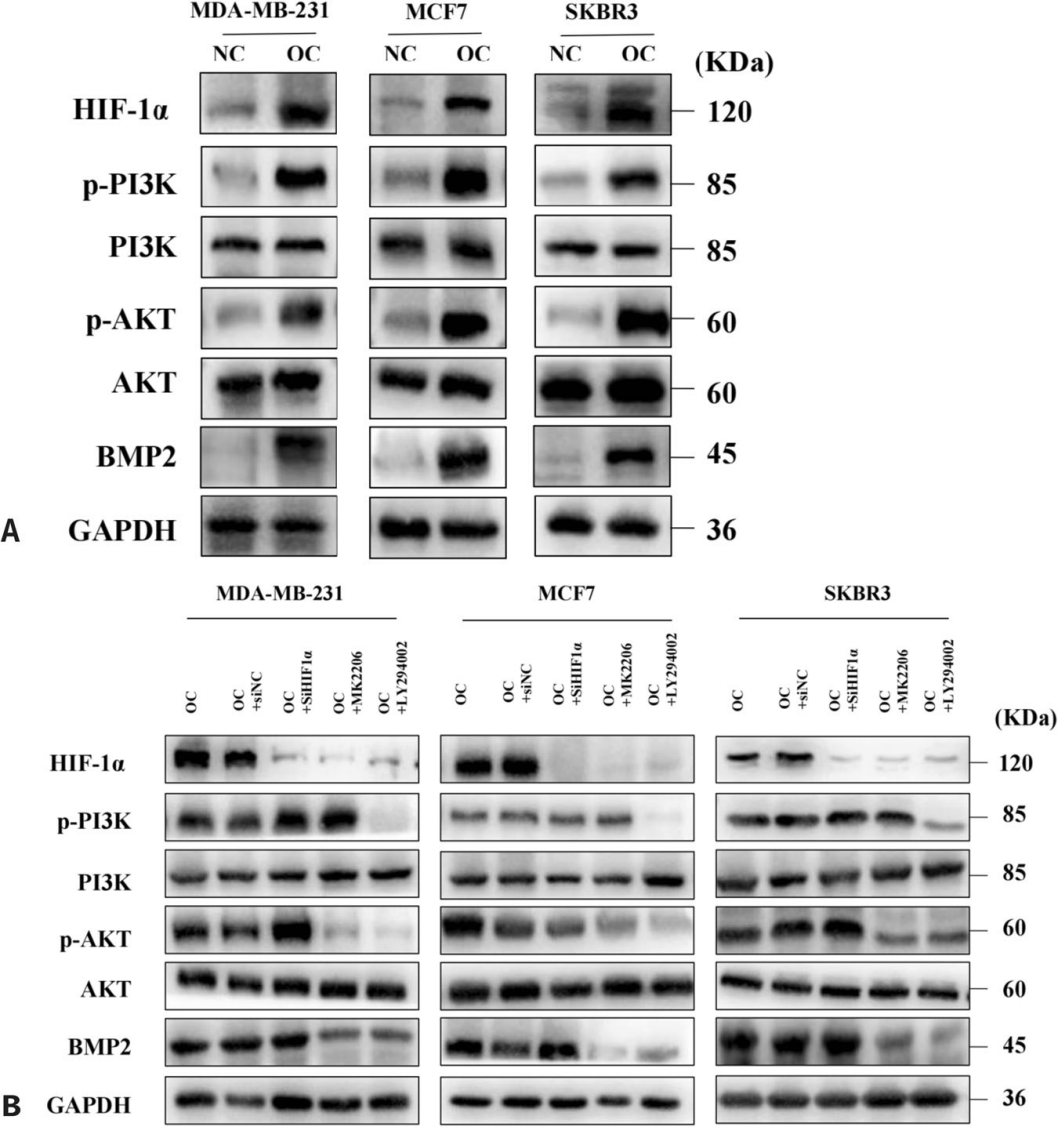
PI3K/AKT signaling pathway upregulates HIF1α expression in calcified cancer cells. **A** Western blots of HIF1α, Bone morphogenetic protein 2 (BMP2), and PI3K/AKT pathway members from normal and calcified cancer cells, GAPDH as internal reference; **B** W\western blots of HIF1α, BMP2, and PI3K/AKT pathway members from cancer cells treated with LY294002 (20 μM, 24 hours), MK2206 (5 μM, 24 hours) or siRNA HIF1α, GAPDH as internal reference.

PI3K/AKT signaling pathway is associated with a variety of cellular processes and plays a major role in fundamental cellular activities such as glucose metabolism, cell metabolism, cell proliferation, cell apoptosis, and angiogenesis. The phosphorylation of PI3K and AKT was significantly increased in the three calcified IBC cells, without changes in the total protein levels of PI3K and AKT. At the same time, we also observed that the expression of BMP2 protein related to calcification is prominently increased (Fig. 4A). This showed that we not only successfully constructed the calcification model of IBC cell lines, but also saw the activation of the PI3K/AKT pathway in this model.

### HIF1α is significantly increased in calcified breast cancer cells and involved in regulating cell activity and glycolytic metabolism

The data of qRT-PCR and Western blotting showed that HIF1α gene and protein levels obviously increased during the formation of calcification, indicating that it played a vital role in the development of IBC with calcification (Figs. 2B, 4A). To further verify the role of HIF1α in calcified breast cancer cells, we transfected HIF1α siRNA in three cell lines treated with OC for 48 hours, provided HIF1α siRNA with liquid change every three days, detected the expression of HIF1α gene and protein levels 72 hours after transfection, and performed cell function experiments. The results showed that HIF1α siRNA stably reduced endogenous HIF1α (Figs. 2B, 4B). The results of cell function experiments showed that IBC cells treated with OC decreased their proliferation and migration ability after inhibiting the expression of HIF1α (Fig. 3A–C). Based on this, we concluded that the HIF1α expression level could affect the malignant potentials of calcified breast cancer cells.

HIF1α is a main regulator of glycolytic activity. It upregulates the expression of genes that encode membrane transporters and glycolytic enzymes^26,27^. Therefore, we simultaneously examined the mRNA expression of enzymes involved in glucose metabolism in IBC cells. The mRNA levels of the glycolytic enzymes, for example, hexokinase 2 (HK2), Glucose Transporter 1 (GLUT1), pyruvate kinase M2 (PKM2), and glucose-6-phosphate dehydrogenase (G6PD), were significantly upregulated in the calcified IBC cells (Fig. 2C). HIF1α knockdown in OC-treated cells decreased cellular glycolysis, and the mRNA expressions of the glycolysis enzymes were significantly decreased (Fig. 2D). These results suggested that metabolic reprogramming with elevated glycolysis may occur in calcified IBC cells and that HIF-1 α was involved in regulating this process.

### PI3K/AKT signaling is involved in breast cancer calcification formation and regulates the activity of calcified breast cancer cells through HIF1α

**Fig. 5:**
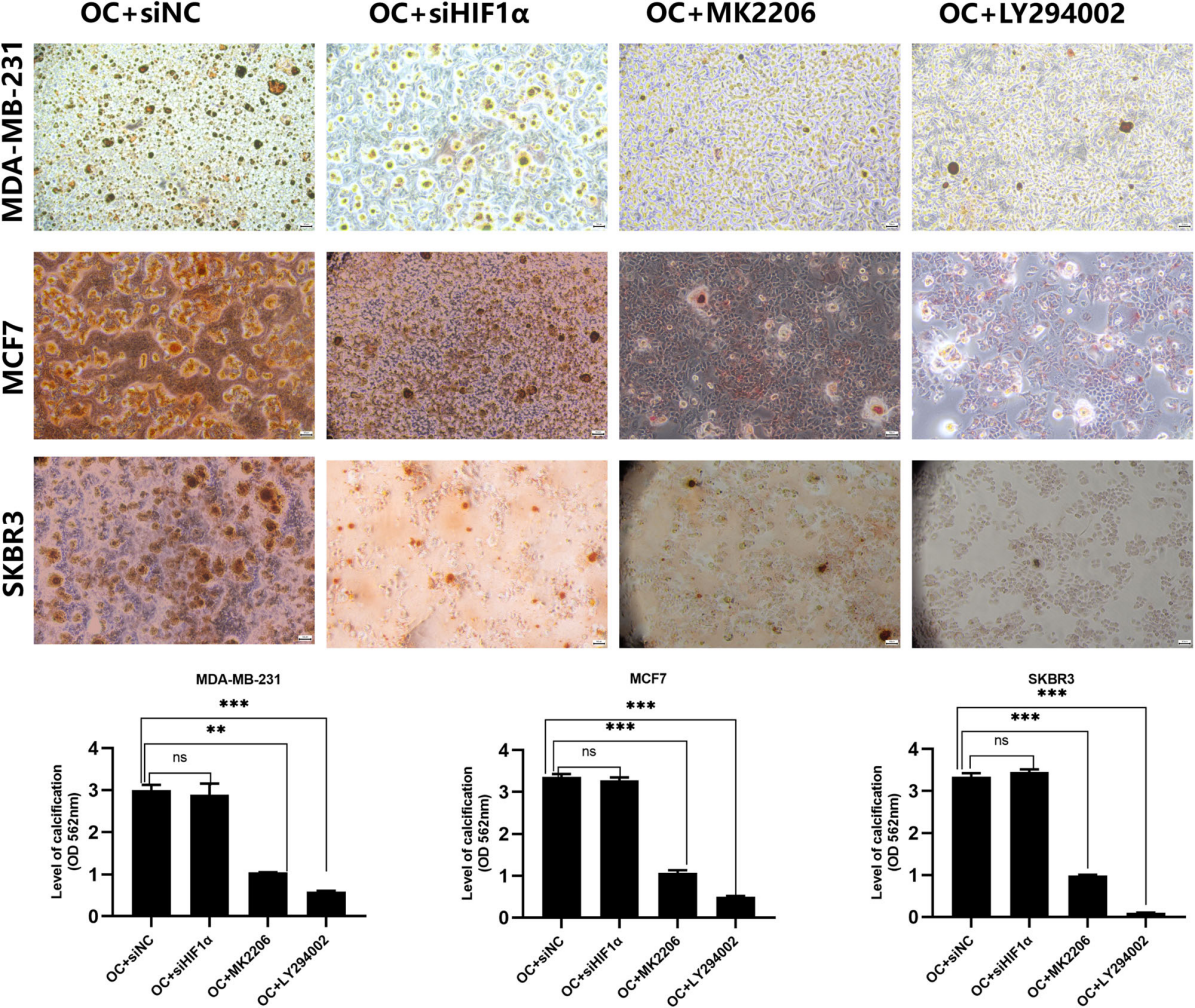
The qualitative and quantitative analysis of calcification when PI3K/AKT pathway was inhibited or HIF1α was knock downed. Data were analyzed by Student’s t test. Remarks: ns *p* ≥ 0.05, **p < 0.01, ***p < 0.001; Data are presented as mean± SD. Bar: mean value; error bar: standard error. Scale bars = 50 µm.

In this study, we initially found the important roles of PI3K/AKT signaling and HIF1α in calcified breast cancer cells. However, the relationship between them still needs further experimental verification. Therefore, LY294002 (PI3K inhibitor) or MK2206 (AKT inhibitor) was added in the mineralization model for 24 hours, and we found that p-PI3K and p-AKT levels were obviously decreased in cells, accompanied with significant reductions of HIF1α protein and mRNA levels (Figs. 4B, 2B). This suggested that the activated PI3K/AKT pathway can upregulate HIF1α expression in cells. However, no significant changes in p-PI3K and p-AKT levels were observed in cells with HIF1α knockdown (Fig. 4B). This indicated that there is no feedback regulation between HIF1α and PI3K/AKT signaling. The qualitative and quantitative analysis of calcification for above-treated cells through alizarin red S and 10% cetylpyridinium chloride showed that inhibition of PI3K/AKT signaling reduced the formation of calcification in cells, whereas knockdown of HIF1α had no effect on calcification (Fig. 5). The occurrence of BMP2 protein is often closely related to the formation of calcification, and its western blotting results were consistent with the results of the calcification analysis (Fig. 4). Together, these results revealed that high expression of HIF1α in OC-induced IBC cells is partly mediated through PI3K/AKT pathway activation. HIF1α was a key regulator of calcified cell activity, but it is not involved in regulating the formation of calcification in IBC cells.

### HIF1α is a key regulator of doxorubicin resistance in calcified breast cancer cells

**Fig. 6:**
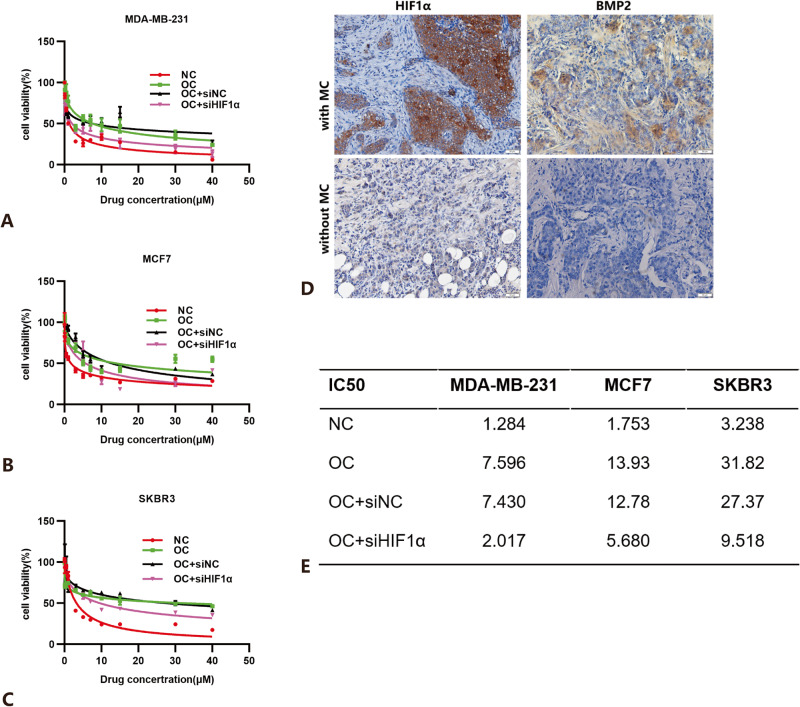
Effect of calcification on doxorubicin chemotherapy sensitivity. **A**–**C** Effect on doxorubicin sensitivity as evaluated by CCK8 assay. MDA-MB-231 cell lines (**A**), MCF7 cell lines (**B**), and SKBR3 cell lines (**C**) were treated with OC and OC-treated cancer cells were transfected HIF1α siRNA; **D** immunohistochemical analysis of HIF-1α and BMP2 in patients receiving doxorubicin neoadjuvant chemotherapy. Scale bars = 50 µm. **E** The IC50 of doxorubicin in three human breast cancer cells. Data were analyzed by Student’s *t* test. Data are presented as mean± SD. Bar: mean value; error bar: standard error.

We investigated the role of calcification in doxorubicin resistance in IBC patients. We collected a total of 109 patients receiving doxorubicin neoadjuvant chemotherapy, of which 57 were without calcification (14 (24.14%) pCR) and 52 with calcification (5 (9.62%) pCR). There is a statistically significant difference between the two groups(*P* = 0.046) (Table 1). From a clinical point of view, IBC patients with calcification were more likely to show doxorubicin resistance, but further experimental verification was needed. We also examined protein expressions, including HIF1α and BMP2, in surgical specimens from patients who received neoadjuvant chemotherapy but did not reach pCR. The results showed that both HIF1α and BMP2 protein expression levels were elevated in IBC patients with calcification. This indicated that HIF1α expression levels may be related to doxorubicin sensitivity in breast cancer patients (Fig. 6D).

**Table 1:** Clinicopathological characteristics of patients with doxorubicin neoadjuvant chemotherapy.

Characteristics	Miller-Payne分级					*P* value
	G1 (*n* = 10)	G2 (*n* = 17)	G3 (*n* = 40)	G4 (*n* = 18)	G5 (*n* = 24)	
Age, year	38.70 ± 7.20	44.41 ± 13.27	47.55 ± 10.95	49.28 ± 7.61	49.04 ± 9.32	*P* = 0.055
ER^**+**^ (%)	8 (80.00)	9 (52.94)	26 (65.00)	10 (55.56)	7 (29.17)	***P*** = **0.031**
PR^+^ (%)	6 (60.00)	3 (17.65)	23 (57.50)	6 (33.33)	9 (37.50)	***P*** = **0.040**
HER2^**+**^ (%)	2 (20.00)	6 (35.29)	11 (27.50)	4 (22.22)	13 (54.17)	*P* = 0.128
Ki67 ≥ 20%(%)	5 (50.00)	9 (52.94)	26 (65.00)	8 (44.44)	17 (70.83)	*P* = 0.385
ANM (%)	8 (80.00)	17 (100.00)	28 (43.08)	8 (44.44)	5 (20.83)	***P*** < **0.001**
pCR, *n* (%)	0 (0.00)	0 (0.00)	0 (0.00)	0 (0.00)	19 (79.17)	***P*** < **0.001**
With MC	7 (70.00)	7 (41.18)	25 (62.50)	6 (33.33)	7 (29.17)	***P*** = **0.032**

*ANM* axillary node metastasis, *MC* microcalcification.

Bold value indicates statistical significance. Data were analyzed by ANOVA and chi-square test.

To further verify our hypothesis, we performed a doxorubicin resistance experiment using the constructed cellular calcification model. In our doxorubicin resistance experiment, the IC50 of normal medium cultured MDA-MB-231, MCF7, SKBR3 were 1.284, 1.753, and 3.238 respectively, while the IC50 of calcified MDA-MB-231, MCF7, SKBR3 were 7.596, 13.93, 31.82. Obviously, IC50 increases significantly. This indicated that calcification did increase the resistance of breast cancer cells to doxorubicin to some degree. To explore the role of HIF1α in doxorubicin resistance, we utilized HIF1α siRNA to decrease HIF1α expression in calcified IBC cells, siNC + OC as a negative control group. Compared with the control group, the HIF1α knockdown group increased doxorubicin susceptibility and decreased IC50, but it was still higher than that in the no-calcification group (Fig. 6A–C, E). Based on this, it was enough to believe that HIF1α expression levels could affect doxorubicin sensitivity in calcified IBC cells. It may even be a key regulator of doxorubicin resistance.

## Discussion

Hydroxyapatite (HA) is the main malignant component of MC in IBC. The osteogenic cocktail used in the research to produce calcification containing HA, which has become a famous model in vitro for calcification researches^28,29^. The data suggests that interaction between cells and HA is related to the colonization and proliferation of IBC cells^30^. The data also shows that the deposited HA might be able to stimulate the migration of surrounding cells in the tumor microenvironment, promote the development of breast cancer, and increase the metastasis of breast cancer cells to distant organs, which were consistent with the poor prognosis of clinical calcified IBC^31,32^. HA can alter the secretion of OPN and OCN, which regulate bone cell signaling with a strong affinity for HA^33–35^. Through repeated experiments, we successfully constructed a cell MC model in three breast cancer cell lines: MDA-MB-231, MCF7, and SKBR3, only taking 48 hours with inorganic phosphate. This is very critical for the exploration and repeated verification of further experiments. Moreover, the results of cell function experiments showed that the invasion and proliferation ability of calcified IBC cells is significantly increased, which fully indicated that MC played a vital role in the development of IBC.

Studies suggest that the formation process of MC in vivo is an active cellular process related to the phenotypic transformation into the mineralized state of breast cancer cells^36–39^. This hypothesis is supported by extensive histopathological studies which show that altered expression of mineralization-related proteins in IBC with MCs, including BMP2, OPN, RUNX2, and ALP^14,40–42^. Other studies have revealed that several calcification-related genes may be related to bone metastasis^43–45^. These genes are potential contributors to the formation of MC, previously confirmed to affect tumor behavior, possibly participating in the formation of MC with increased tumor invasion. Alterations of these genes were also observed in our experimental model, proving that our calcified cell model supports the hypothesis that MC is similar to the mineralization process that occurs during osteogenesis.

The activated PI3K/AKT pathway plays a key role in a variety of cancers (including IBC), especially the regulation of substance and energy metabolism^46–48^. Previous related studies have revealed that PI3K/AKT pathway is associated with the formation of MCs in IBC^8,42,49^. Regardless of oxygen levels, activated PI3K/AKT signaling upregulates the transcription and translation of HIF1α and increases cellular glycolysis^50,51^. The enhanced stability and transcription of HIF1α have been broadly reported in various tumors undergoing malignant progression^52^. All the time, HIF1α has been considered the main coordinator of cellular adaptation to the oxygen environment, but it has been shown that some non-classical regulation of HIF1α is not affected by external oxygen concentration^53^. In the study, we observed a high expression of HIF1α in calcified breast tissues and cells. In OC-treated breast cancer cells, HIF1α mRNA and protein are regulated by PI3K/AKT signaling, not in response to oxygen conditions. The activation of HIF1α participates in the transcription of genes involved in important cancer biology, including cell survival, angiogenesis, glucose metabolism, and invasion^27,54,55^. It has been shown that HIF1α induces the generation of vascular calcification through regulating glucose metabolism^56–59^. In some cases, altered metabolism can regulate Epithelial to mesenchymal transition in tumor cells^60,61^. Malignant tumor cells maintain their proliferation and metastatic potential by enhancing aerobic glycolysis^55,62^. In our calcified model, we also observed that mRNA levels of enzymes involved in glycolysis were significantly increased in calcified cells, and this elevated trend disappeared after knockdown of HIF1α. Meanwhile, with the decreased expression of HIF1α, the proliferation and aggression of cells were also notably decreased. This indicates that in calcified IBC cells, the activation of PI3K/AKT pathway not only participates in the generation of MC, but also further activates the stable expression of its downstream HIF1α, thus increasing cell proliferation and invasion ability through the alteration of cell glucose metabolism. Interestingly, it was unexpectedly found that when HIF1α was knocked down, calcified cancer cells could still produce calcification, but their proliferation and invasive ability were significantly decreased. Thus, we guess that calcification itself may not affect the malignant potential of cancer cells. But in the process of calcification formation, its key pathway PI3K/AKT is activated, leading to the activation of a series of downstream factors, such as HIF1 α explored in the study, which is a key regulator of glycolysis, thus induces the metabolic reprogramming of cells, increases the cell invasion and proliferation ability. This provides research ideas for IBC treatment with calcification, and more focus on downstream factors than calcification itself may be more valuable. Of course, this is different from the conclusions of some studies, which suggest that HA itself may have biological effects on the surrounding cells to promote the progression of breast cancer^30,31,63–65^ ③. Perhaps deeper research in vivo is likely to help us resolve this contradiction.

Studies showed that the presence or absence of calcification was not associated with the neoadjuvant chemotherapy effect in triple-negative breast cancer^66^. However, other studies associated MC with pCR after neoadjuvant chemotherapy in breast cancer with HER2 overexpression^67^. In others, IBC with calcification patients receiving neoadjuvant chemotherapy were more likely to have disease progression and had lower pCR response rate^68^. Anthracyclines are widely used in neoadjuvant and adjuvant chemotherapy for IBC^69,70^. In the cases collected, we found that the pCR of patients without calcification in doxorubicin neoadjuvant chemotherapy was 31.04%, which was higher than that of IBC patients with calcification (11.76%). This suggests that MC in breast cancer has a negative effect on chemotherapeutic drugs and may induce resistance to anthracyclines. Meanwhile, the results of our doxorubicin resistance experiment showed that the IC50 of OC-treated MDA-MB-231, MCF7, SKBR3 cells significantly increased, and decreased after HIF1α knockdown. Therefore, based on above experimental results, we preliminarily believe that the PI3K/AKT pathway may promote HIF1α-mediated reprogramming of glucose metabolism to induce doxorubicin resistance in IBC with MC.

Unfortunately, we failed to construct a valid xenograft model of calcification in vivo using human breast cancer cells. There are few known animal models of calcified IBC. Most of them involve exogenous gene interference, which is unfavorable for subsequent studies^71–73^. Although the direct translation of experimental results from cell lines into tumors remains a controversial issue, the study represents a starting point for previously under-explored areas. Perhaps building models of animal calcification in a stable natural state could help verify our hypothesis, which could be the focus of our next stage.

Taken together, We successfully constructed the rapidly calcified model using inorganic phosphate in MDA-MB-231, MCF7, SKBR3 IBC cell lines. This is very favorable for subsequent studies related to calcification in IBC. In the study, we found that MC itself was only a symptom. When the HIF1α expression is blocked, as the downstream of PI3K/AKT pathway, the invasion and proliferation of cells are significantly decreased even though calcification still exists. So only the series of chain reactions stimulated during calcification formation deserve our further attention. We also attempted to conduct doxorubicin resistance experiment with the calcified model, and successfully found the vital regulator HIF1α, which may be an effective therapeutic target in clinical treatment. We also provide preliminary data on the potential value of HIF1α as a biomarker for doxorubicin resistance. Overall, the MC is a poor prognostic factor in IBC patients, and our findings provide a new direction for exploring the MC in IBC.

## Methods

### Cell culture

MCF7 (catalog number HTB-22, ATCC), MDA-MB-231 (catalog number CRM-HTB-26, ATCC), and SKBR3(catalog number HTB-30, ATCC) cell lines were obtained from American Type Culture Collection (http://www.atcc.org). MDA-MB-231 and MCF7 cell lines were cultured using DMEM medium which contains 10% fetal bovine serum (FBS) (Gibco), while SKBR3 needs 15% FBS. Mycoplasma contamination was tested using PlasmoTest Mycoplasma Detection Kit (InvivoGen), and no contamination was ensured. All cell lines were cultured at 37 °C in a humidified incubator with 5% CO_2_. All purchased cell lines were STR-identified and compared to authoritative databases.

### Cell mineralization in vitro

Cells were seeded into six-well plates and cultured using a regular medium at a cell density of (2–4)× 10^5^ cells/mL (day 0). The following day (Day 1), cells were cultured with calcified medium consisting of 50 μg/mL ascorbic acid (Sigma-Aldrich # A4403), 10 mM inorganic phosphate (Sigma-Aldrich, #71496), and 10–100 nM dexamethasone (Sigma-Aldrich, # D4902). All cells were incubated in a calcified medium for 3, 5, 7, and 14 days, with a change of medium every 2–3 days to evaluate the calcification. The control group was cultured using the conventional medium.

### Mineralization evaluation

Calcification and quantification of calcification were evaluated by Alizarin Red S staining and von Kossa staining. Remove the medium in the six-well plates and wash the cells three times with phosphate-buffered saline (PBS) (Servicebio). Then fix the cells with 4% paraformaldehyde (Servicebio) for 30 min and wash again with PBS three times for 5 min. Finally stain with Alizarin Red S (2%, pH 4.4) (Servicebio) for 10 minutes and rinse in PBS until colorless. Imaging was performed under an optical microscope, and red staining indicates a positive calcification result. Finally, PBS was incubated for 15 min to remove non-specific binding. PBS in the six-well plate was discarded, then 400 μL of 10% cetylpyridinium chloride (MCE) was added to each well, stand for 30 min. Finally, the microplate reader wa used to measure absorbance value of per well at 562 nm.

Results of Alizarin Red S staining were verified using von Kossa staining kit (Servicebio) with a separate six-well plate. Firstly, The cells were fixed with 4% paraformaldehyde for 30 min, then incubated with 5% silver nitrate solution for 30 min at room temperature, washed three times, placed under Ultraviolet light for 30 min, and subsequently counterstained with eosin for 30 sec. Six-well plates were dried and imaged under an light microscope, dark brown or black indicating positive calcium phosphate results.

### Quantitative real-time fluorescence PCR

Total RNA was isolated using Trizol reagent (Invitrogen) and reverse transcribed using a reverse transcription kit (Invitrogen). qRT-PCR was performed using a SYBR Green premix (ABgene) and the primers were performed using a real-time PCR detection system (Bio-Rad). The expression of genes was normalized to the expression of GAPDH, and 2^−ΔΔCt^ method was used to analyze the results. Primer sequences are shown in Table 2.

**Table 2:** Primer sequences utilized for qRT-PCR analysis.

Target	Forward primer	Reverse primer
GAPDH	GGAGCGAGATCCCTCCAAAAT	GGCTGTTGTCATACTTCTCATGG
BMP2	ACCCGCTGTCTTCTAGCGT	TTTCAGGCCGAACATGCTGAG
OCN	GGCGCTACCTGTATCAATGG	GTGGTCAGCCAACTCGTCA
OPN	CTCCATTGACTCGAACGACTC	CAGGTCTGCGAAACTTCTTAGAT
RUNX2	CCGCCTCAGTGATTTAGGGC	GGGTCTGTAATCTGACTCTGTCC
ALP	ACTGGGGCCTGAGATACCC	TCGTGTTGCACTGGTTAAAGC
PFKP	GACCTTCGTTCTGGAGGTGAT	CACGGTTCTCCGAGAGTTTG
GLUT1	TCTGGCATCAACGCTGTCTTC	CGATACCGGAGCCAATGGT
GLUT4	ATCCTTGGACGATTCCTCATTGG	CAGGTGAGTGGGAGCAATCT
G6PD	CGAGGCCGTCACCAAGAAC	GTAGTGGTCGATGCGGTAGA
HK1	CACATGGAGTCCGAGGTTTATG	CGTGAATCCCACAGGTAACTTC
HK2	TTGACCAGGAGATTGACATGGG	CAACCGCATCAGGACCTCA
MCT1	AGTAGTTATGGGAAGAGTCAGCA	GTCGGGCTACCATGTCAACA
MCT4	AGGTATCCTTGAGACGGTCAG	CAAGCAGGTTAGTGATGCCG
ENO1	GCCGTGAACGAGAAGTCCTG	ACGCCTGAAGAGACTCGGT
PKM2	ATGTCGAAGCCCCATAGTGAA	TGGGTGGTGAATCAATGTCCA
PDK1	GAGAGCCACTATGGAACACCA	GGAGGTCTCAACACGAGGT
LDHA	ATGGCAACTCTAAAGGATCAGC	CCAACCCCAACAACTGTAATCT

### siRNA knockdown

HIF1α siRNA (siHIF1α) and negative control siRNA (siNC) were synthesized by RiboBio (Ribobio, Guangzhou, China). siHIF1α was transfected into breast cancer cells using Lipofectamine transfection reagent (Invitrogen) according to the manufacturer’s instructions when the cell density was at 70–80%. Cells transfected with siNC were used as controls. Transfected cells were harvested 72 hours after transfection. The siRNA sequences are stated in the Supplementary information.

### Inhibition of the PI3K/AKT signaling pathway

MDA-MB-231, MCF7, and SKBR3 cells were seeded into six-well plates with a cell density of (2–4) × 10^5^ cells/ml, and the medium containing OC was added for 48 hours after being cultured for 24 hours. After the fluid changed, PI3K inhibitor LY294002 (Merck Biosciences) 20 μM, AKT inhibitor MK2206 (Merck Biosciences) 5 μM were added to the culture medium for 24 hours. For the control groups, a culture medium containing 0.04% dimethyl sulfoxide (Sigma) was used. Protein blots were performed on day 7, stained with Alizarin Red S, and quantitative calcification determination by 10% cetylpyridinium chloride.

### Western blotting

Collect cells and extract protein on ice with cold RIPA lysis buffer (Servicebio) containing protease inhibitors. Quantify the protein concentration, separate protein by gel electrophoresis, and transblot onto a polyvinylidene difluoride membrane (Merck Millipore) which were incubated together with the primary antibodies at 4 °C overnight. Then incubate the membranes with dilutions of the HRP-conjugated secondary antibody for one hour at room temperature. Protein expression was detected using a hypersensitivity ECL test kit (Thermo Fisher Scientific, Norwalk, Connecticut, USA). Images were obtained on a film processor. GAPDH was used as a control. The primary antibodies were listed as follows: p-PI3K (T40065S, abmart, China, 1:1000), PI3K (T56915, abmart, China, 1:1000), p-AKT (T40067S, abmart, China, 1:1000), AKT (T55561, abmart, China, 1:1000), BMP2 (66383-1-Ig, proteintech, China, 1:1000), HIF1α (#14179, CST, USA, 1:1000), GAPDH (ANT 325, AntGene, China, 1:5000).

### Detection of cell proliferation

CCK8 and colony formation assay were applied for evaluation of cell proliferation. Three untreated breast cancer cells and cells treated with OC for 48 hours were seeded at a density of 2500 cells/well into 96-well culture plates at the condition of 37 °C and 5% CO_2_, and then cultured with DMEM medium for 1–4 days. The cells were then incubated in DMEM medium containing 10% CCK8 (Cell Counting Kit-8) solution for 1 hour. The absorbance was measured at 450 nm, and the data of cell proliferation curves were plotted. The assay was repeated at least three times. For colony formation, the above cells were seeded into six-well plates at a density of 2000 cells/well, and after 7 days of incubation, cells on six-well plate were fixed with crystal violet (ServiceBio) and finally calculated by Image J software.

### Scratching

In the wound-healing assay, 70 μL cells were seeded at a density of 5 × 10^5^ Cells/mL in IBIDI two-well culture (Ibidi) inserts placed in 24-well plates. Complete confluence of cells in the well was observed under the light microscope, and then the inserts were carefully removed with tweezers in a clean bench. Cells were gently washed three times with PBS, 1 mL DMEM medium containing 1% FBS was added to each well, and cell migration was photographed at 0 hour and 48 hours under the light microscope.

### Transwell

The cells with a density of 1 × 10^4^cells/mL were added to the top chamber plate of the transport well (Thermo Fisher Scientific), and cultured for 24 hours using 200 μL DMEM medium without FBS. 500 μL DMEM medium containing 15% FBS was used as a chemoattractant. Fix the migration cells with 4% paraformaldehyde at normal temperature for 15 min, crystal violet solution for 15 min, and gently wash the cells with PBS. The cells above the indoor microporous film were carefully wiped with a cotton swab. The microporous film was dried, cut, and put on the glass slide, sealed by cover glass and neutral resin, observed, and photographed under the optical microscope.

### Study population

From December 2020 to June 2022, a total of 109 IBC patients who received neoadjuvant chemotherapy with doxorubicin were randomly admitted to the Tongji Hospital of Huazhong University of Science and Technology. Clinical information was collected from patient medical records, which include age, menstrual status, tumor size, molecular typing, axillary node metastasis (ANM), and histological grade. According to the presence or absence of MC in the breast X-ray photography, patients were divided into two groups. Patients with previous doxorubicin treatment, other malignancies, hypercalcemia, hyperparathyroidism, or renal failure were excluded. The research was permitted by the Institutional Review Board of Tongji Hospital (Wuhan, China) (TJ-IRB20221137). All participants have signed an informed consent form.

### Immunohistochemistry (IHC)

Tissue samples were collected from patients during surgery after neoadjuvant chemotherapy. The expressions of BMP2 and HIF1α in paraffin-embedded tissues were detected by immunohistochemistry. In this study, all samples were fixed in 4% paraformaldehyde and then cut into 4 μm slices after paraffin embedding, dewaxed in xylene, blocked with 3% hydrogen peroxide, washed with PBS, and then blocked by goat serum at room temperature. Specimens were mixed with anti-HIF1α primary antibody (rabbit polyclonal, 20960-1-AP, Proteintech, China, 1:50) or BMP2 (murine monoclonal, 66383-1-AP, Proteintech, Wuhan, China, 1:50) at 4 °C overnight. Next, the slices were incubated with Horseradish peroxidase (HRP) plus goat anti-mouse/rabbit IgG (PR30012/PR30011, Proteintech, 1:5000) for 30 min at normal temperature. The diaminobenzidine (DBA) (Proteintech) was treated for 5 to 10 min. Then they were counterstained by hematoxylin, dehydrated with alcohol and xylene, and mounted. Finally, the sections were sealed with a neutral glue. All subjects signed an informed consent. All studies adhered to the Declaration of Helsinki.

### Doxorubicin sensitivity experiment

Trypsin (ServiceBio) digested the cells to prepare cell suspensions and cell counts were performed under a microscope. In 96-well plates, 5000 cells were seeded per well. After incubation of 12 hours, doxorubicin (MCE) was added according to the concentration gradient of 0, 0.1, 0.5, 1, 3, 5, 7, 10, 15, 30, 40 μM/L. Each concentration was repeated three times. After incubation of 24 hours, remove the fluid in the 96-well plates, then 10 μL CCK-8 reagent and 90 μL DMEM medium were added to each well and three cell-free blank groups were set. The cells were cultured at a 37 °C incubator for one hour, then measured the absorbance (OD) at 450 nm. The cell viability was calculated according to the formula: cell viability (%) = (experiment OD-blank OD)/(control OD-blank OD). Graphpad Software was used to calculate the IC50.

### Statistical analysis

GraphPad Prism 8 software was used for statistical analyses. Categorical variables were analyzed by the chi-square test. Continuous variables are presented as the mean ± SD or medians with interquartile range, analyzed by Student’s *t* test or one-way analysis of variance (ANOVA). *P* < 0.05 was considered statistically significant. Experiments were repeated at least three times each time in triplicate.

### Reporting summary

Further information on research design is available in the Nature Research Reporting Summary linked to this article.

### Supplementary information

Supplementary Material

Reporting Summary

## Data Availability

All data generated or analyzed during the research are already included in the paper. Datasets supporting the table and data for IHC in this article are not available for the purpose of protecting patient privacy but can be accessed from the corresponding author on request.
