# Supplementary material for: PI3K/AKT signaling activates HIF1α to modulate the biological effects of invasive breast cancer with microcalcification
Source: NPJ Breast Cancer. 2023 Nov 13;9:93. doi: 10.1038/s41523-023-00598-z (PMC10643473; doi:10.1038/s41523-023-00598-z)
Supplement: Supplementary file 1 — Supplementary Material [file 41523_2023_598_MOESM1_ESM.pdf]

siRNA sequences

si-NC:

5’-TTCTCCGAACGTGTCACGTdTdT-3’

si-HIF1α:

5’-GGAACATGATGGTTCACTT-3’

Uncropped and unprocessed scans of blots

Figure 4A

MDA-MB-231

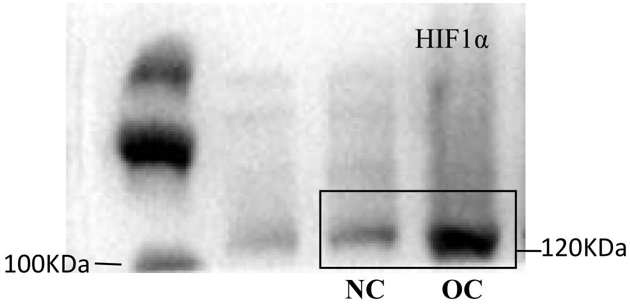

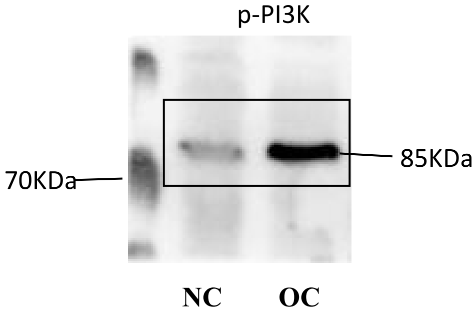

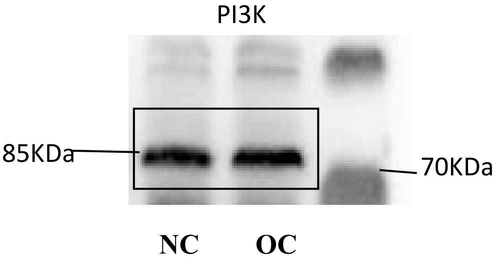

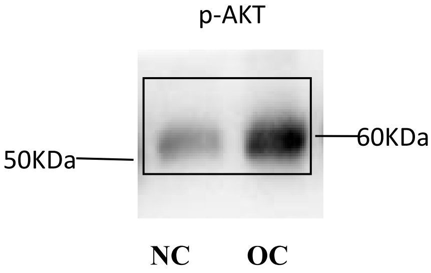

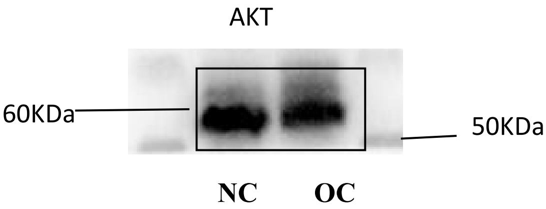

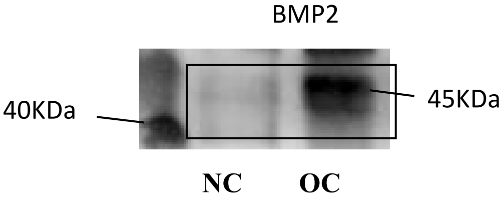

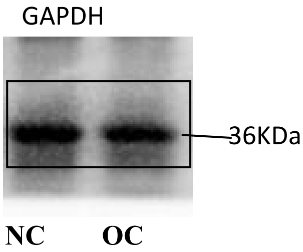

MCF7

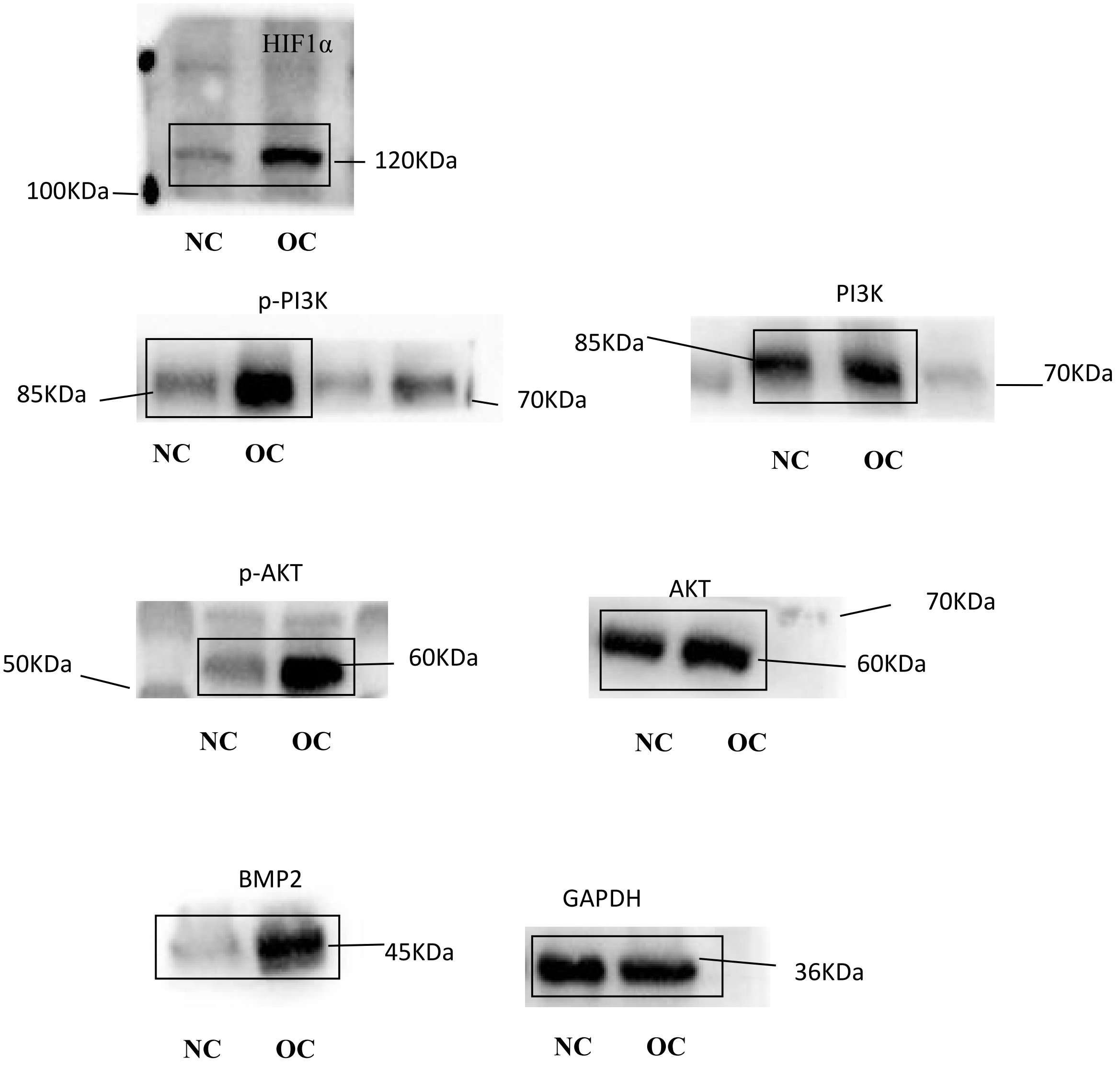
HIF1α
120KDa
100KDa
NC
OC
p-PI3K
85KDa
70KDa
NC
OC
PI3K
85KDa
70KDa
NC
OC
p-AKT
50KDa
60KDa
NC
OC
AKT
70KDa
60KDa
NC
OC
BMP2
45KDa
NC
OC
GAPDH
36KDa
NC
OC

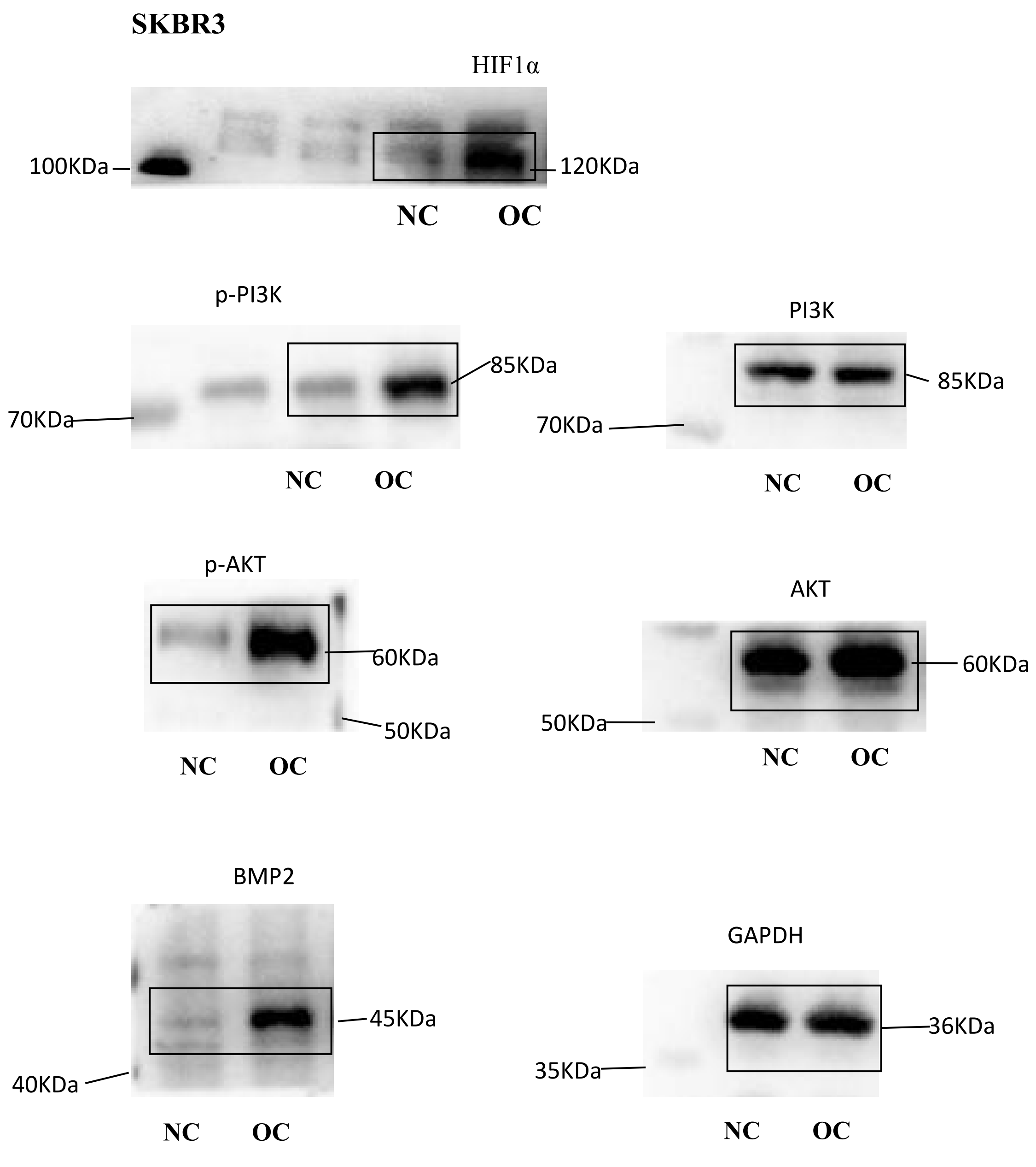
SKBR3
HIF1α
100KDa
120KDa
NC
OC
p-PI3K
70KDa
85KDa
NC
OC
PI3K
70KDa
85KDa
NC
OC
p-AKT
60KDa
50KDa
NC
OC
AKT
50KDa
60KDa
NC
OC
BMP2
45KDa
40KDa
NC
OC
GAPDH
35KDa
36KDa
NC
OC

Figure 4B

MDA-MB-231

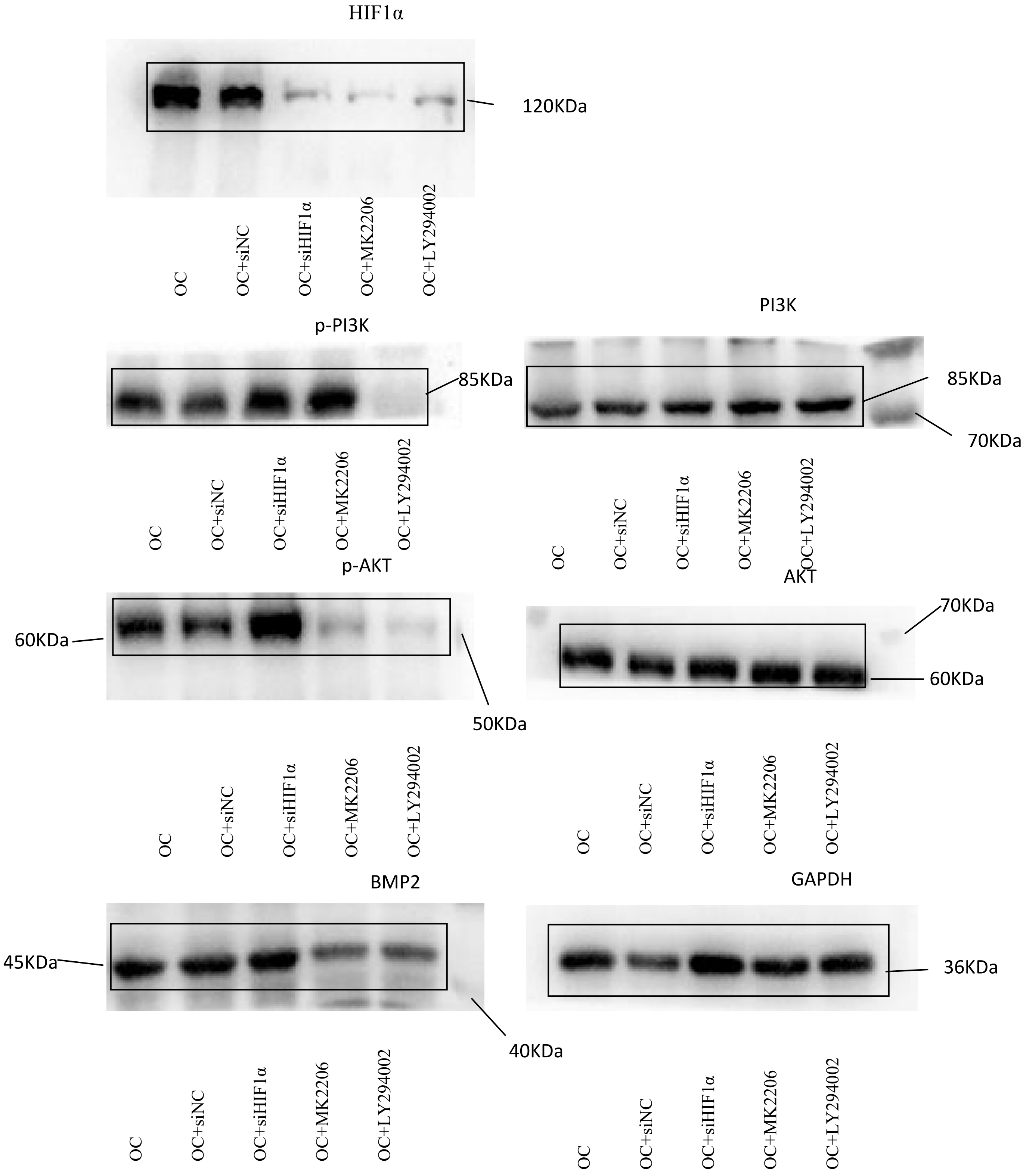

MCF7

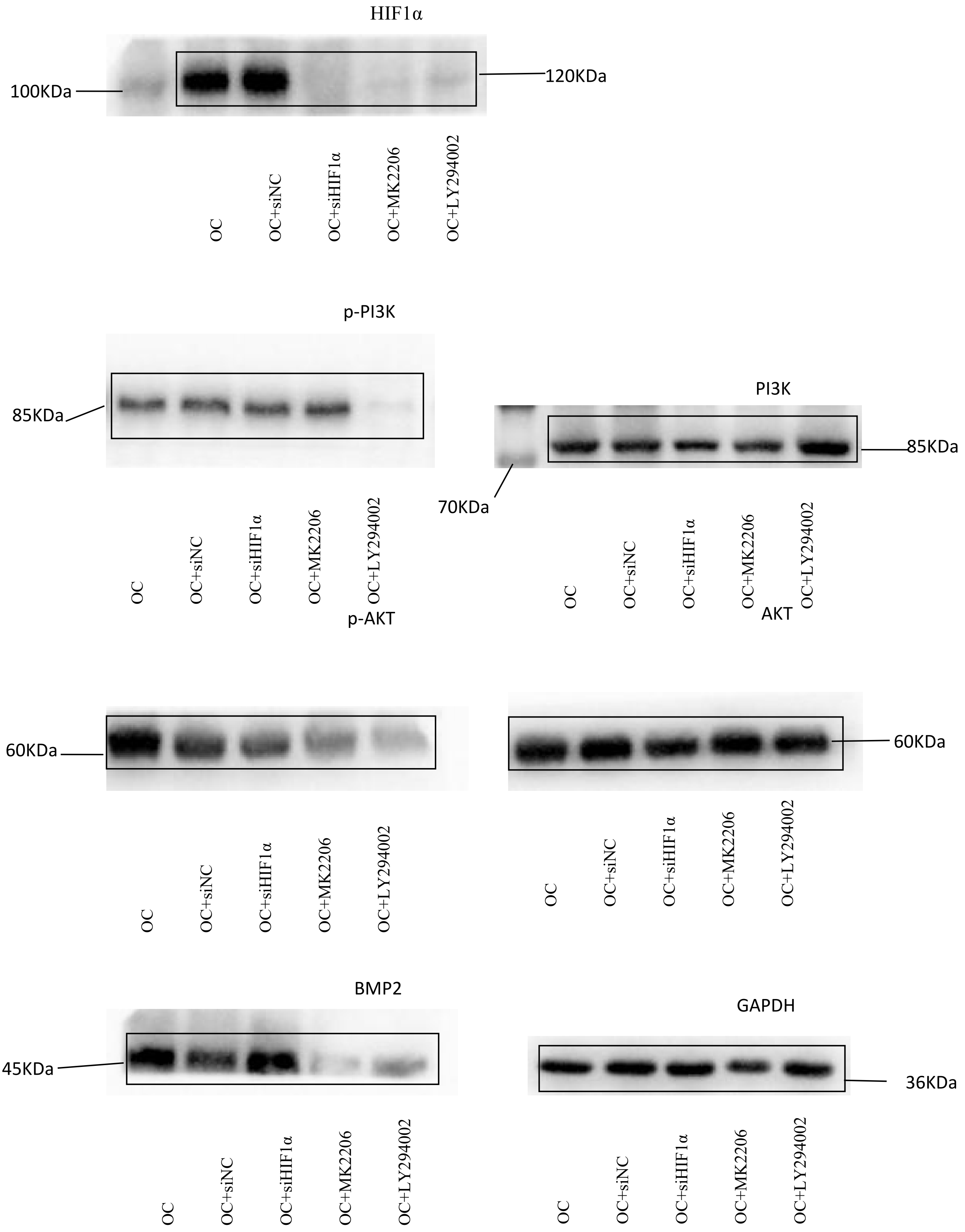
HIF1α
100KDa
120KDa
OC
OC+siNC
OC+siHIF1α
OC+MK2206
OC+LY294002
p-PI3K
85KDa
OC
OC+siNC
OC+siHIF1α
OC+MK2206
OC+LY294002
PI3K
85KDa
70KDa
OC
OC+siNC
OC+siHIF1α
OC+MK2206
OC+LY294002
p-AKT
60KDa
OC
OC+siNC
OC+siHIF1α
OC+MK2206
OC+LY294002
AKT
60KDa
OC
OC+siNC
OC+siHIF1α
OC+MK2206
OC+LY294002
BMP2
45KDa
OC
OC+siNC
OC+siHIF1α
OC+MK2206
OC+LY294002
GAPDH
36KDa
OC
OC+siNC
OC+siHIF1α
OC+MK2206
OC+LY294002

SKBR3

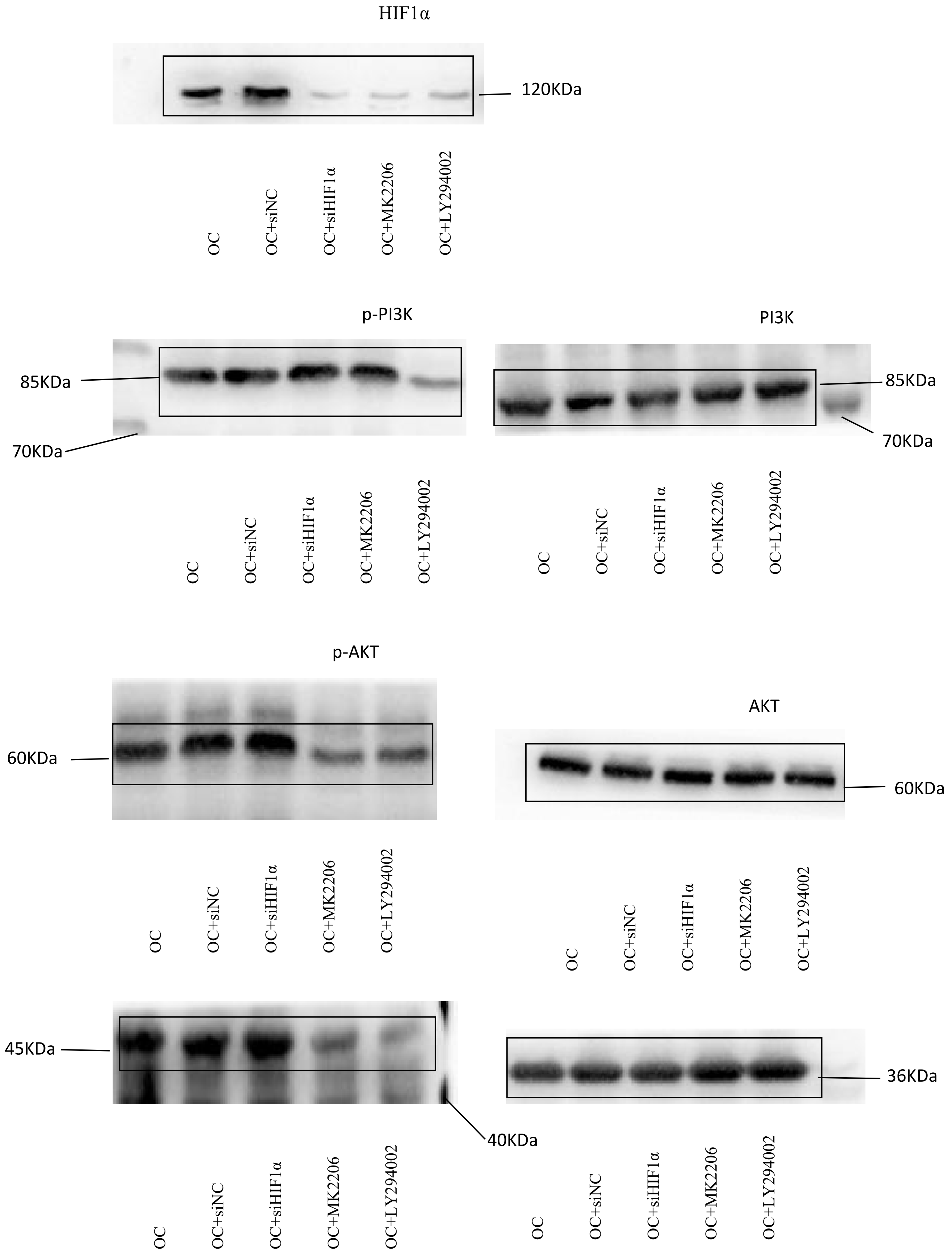

HIF1α
120KDa
OC
OC+siNC
OC+siHIF1α
OC+MK2206
OC+LY294002
p-PI3K
85KDa
70KDa
PI3K
85KDa
70KDa
p-AKT
60KDa
AKT
60KDa
45KDa
40KDa
36KDa
