# Supplementary material for: PI3K/AKT signaling activates HIF1α to modulate the biological effects of invasive breast cancer with microcalcification
Source: NPJ Breast Cancer. 2023 Nov 13;9:93. doi: 10.1038/s41523-023-00598-z (PMC10643473; doi:10.1038/s41523-023-00598-z)
Supplement: Supplementary file 2 — Reporting Summary [file 41523_2023_598_MOESM2_ESM.pdf]

nature portfolio

Corresponding author(s): Lin Zhang

Last updated by author(s): Oct 11, 2023

Reporting Summary

Nature Portfolio wishes to improve the reproducibility of the work that we publish. This form provides structure for consistency and transparency in reporting. For further information on Nature Portfolio policies, see our Editorial Policies and the Editorial Policy Checklist.

Statistics

For all statistical analyses, confirm that the following items are present in the figure legend, table legend, main text, or Methods section.

n/a	Confirmed	
☐	☒	The exact sample size (*n*) for each experimental group/condition, given as a discrete number and unit of measurement
☐	☒	A statement on whether measurements were taken from distinct samples or whether the same sample was measured repeatedly
☐	☒	The statistical test(s) used AND whether they are one- or two-sided<br>*Only common tests should be described solely by name; describe more complex techniques in the Methods section.*
☐	☒	A description of all covariates tested
☐	☒	A description of any assumptions or corrections, such as tests of normality and adjustment for multiple comparisons
☐	☒	A full description of the statistical parameters including central tendency (e.g. means) or other basic estimates (e.g. regression coefficient) AND variation (e.g. standard deviation) or associated estimates of uncertainty (e.g. confidence intervals)
☐	☒	For null hypothesis testing, the test statistic (e.g. *F*, *t*, *r*) with confidence intervals, effect sizes, degrees of freedom and *P* value noted<br>*Give P values as exact values whenever suitable.*
☒	☐	For Bayesian analysis, information on the choice of priors and Markov chain Monte Carlo settings
☒	☐	For hierarchical and complex designs, identification of the appropriate level for tests and full reporting of outcomes
☒	☐	Estimates of effect sizes (e.g. Cohen's *d*, Pearson's *r*), indicating how they were calculated

Our web collection on statistics for biologists contains articles on many of the points above.

Software and code

Policy information about availability of computer code

Data collection	GraphPad Prism 8 software.
Data analysis	GraphPad Prism 8 software

For manuscripts utilizing custom algorithms or software that are central to the research but not yet described in published literature, software must be made available to editors and reviewers. We strongly encourage code deposition in a community repository (e.g. GitHub). See the Nature Portfolio guidelines for submitting code & software for further information.

Data

Policy information about availability of data

All manuscripts must include a data availability statement. This statement should provide the following information, where applicable:

- Accession codes, unique identifiers, or web links for publicly available datasets
- A description of any restrictions on data availability
- For clinical datasets or third party data, please ensure that the statement adheres to our policy

All data generated or analyzed during the research are already included in the paper . Datasets supporting the table and data for IHC in this article are not available on the purpose of protecting patient privacy, but can be accessed from the corresponding author on request.

Research involving human participants, their data, or biological material

Policy information about studies with human participants or human data. See also policy information about sex, gender (identity/presentation), and sexual orientation and race, ethnicity and racism.

Reporting on sex and gender	Breast cancer is the most common cancer among women, so our study population was female.
Reporting on race, ethnicity, or other socially relevant groupings	There was no racial difference in our study population, which was all Asian.
Population characteristics	From December 2020 to June 2022, a total of 109 IBC patients who have received neoadjuvant chemotherapy with doxorubicin were randomly admitted in the Tongji Hospital of Huazhong University of Science and Technology.
Recruitment	Single center study, data conclusions have limitations.
Ethics oversight	The research was permitted by the Institutional Review Board of Tongji Hospital (Wuhan, China) (TJ-IRB20221137). All participants have signed an informed consent form.

Note that full information on the approval of the study protocol must also be provided in the manuscript.

Field-specific reporting

Please select the one below that is the best fit for your research. If you are not sure, read the appropriate sections before making your selection.

☒ Life sciences ☐ Behavioural & social sciences ☐ Ecological, evolutionary & environmental sciences

For a reference copy of the document with all sections, see nature.com/documents/nr-reporting-summary-flat.pdf

Life sciences study design

All studies must disclose on these points even when the disclosure is negative.

Sample size	We selected the cases that appeared in the course of this study, followed the treatment progress of the patients, selected the cases according to strict inclusion criteria, and determined the final sample size.
Data exclusions	No data were excluded from the analyses.
Replication	Experiments were repeated at least three times each time in triplicate. All attempts at replication were successful.
Randomization	Participants data were randomly collected.
Blinding	The investigators were blinded to group allocation during data collection and analysis.

Reporting for specific materials, systems and methods

We require information from authors about some types of materials, experimental systems and methods used in many studies. Here, indicate whether each material, system or method listed is relevant to your study. If you are not sure if a list item applies to your research, read the appropriate section before selecting a response.

Materials & experimental systems

n/a	Involved in the study	
☐	☒	Antibodies
☐	☒	Eukaryotic cell lines
☒	☐	Palaeontology and archaeology
☒	☐	Animals and other organisms
☐	☒	Clinical data
☒	☐	Dual use research of concern
☒	☐	Plants

Methods

n/a	Involved in the study	
☒	☐	ChIP-seq
☒	☐	Flow cytometry
☒	☐	MRI-based neuroimaging

Antibodies

Antibodies used	p-PI3K (abmart, T40065S,1:1000, China), PI3K (abmart, T56915,1:1000, China), p-AKT (abmart, T40067S,1:1000, China), AKT (abmart, T55561, 1:1000, China), BMP2 (proteintech,66383-1-Ig, 1:1000, China), HIF1α (CST, #14179, 1:1000, USA), GAPDH (AntGene,ANT

	325, 1:5000, China), Ultrapolymer Goat anti-Mouse IgG(H&L)-HRP (Proteintech,PR30012,1:5000,China) Ultrapolymer Goat anti-Rabbit IgG(H&L)-HRP (Proteintech,PR30011,1:5000,China)
Validation	p-PI3K (abmart, T40065S,1:1000, China) Isotype:Rabbit IgG, Reactivity: Human Mouse Rat, Recommend Antibody Dilutions: WB 1:500-1:2000 IF/ICC 1:100-1:500<br>PI3K (abmart, T56915,1:1000, China) Isotype:Rabbit IgG, Reactivity: Human Mouse Rat, Recommend Antibody Dilutions: WB 1:500-1:2000<br>p-AKT (abmart, T40067S,1:1000, China) Isotype:Rabbit IgG, Reactivity: Human Mouse Rat, Recommend Antibody Dilutions: WB 1:500-1:2000 IHC 1:50-1:200 IF/ICC 1:100-1:500<br>AKT (abmart, T55561, 1:1000, China) Isotype:Rabbit IgG, Reactivity: Human Mouse Rat, Recommend Antibody Dilutions: WB 1:500~1:2000 IHC 1:50~1:200 ICC/IF 1:50~1:200 IP 1:50<br>BMP2 (proteintech,66383-1-Ig, 1:1000, China) Isotype: Mouse IgG2a, Reactivity: Human Mouse Rat Pig, Recommend Antibody Dilutions: WB : 1:1000-1:4000 IHC : 1:50-1:500<br>HIF1α (CST, #14179, 1:1000, USA) Isotype:Rabbit IgG, Reactivity: Human Mouse Rat Monky, Recommend Antibody Dilutions: WB 1:1000 ChIP-seq 1:50 CUT&RUN:1:50<br>GAPDH (AntGene,ANT 325, 1:5000, China) Isotype:Rabbit IgG, Reactivity: Human Mouse Rat Pig Arabidopsis Corn Cabbage Rice, Recommend Antibody Dilutions: WB : 1:5000-1:40000 IP : 0.5-4.0 ug for IP and 1:1000-1:6000 for WB IHC :1:200-1:800 IF : 1:500-1:2000

Eukaryotic cell lines

Policy information about cell lines and Sex and Gender in Research

Cell line source(s)	MDA-MB-231, source: Hydrothorax from a 51-year-old woman with metastatic breast adenocarcinoma.<br>MCF7, source: Pleural effusion from a 69-year-old white female with breast cancer.<br>SKBR3, source: Pleural effusion from a 43-year-old white female with breast cancer.
Authentication	All purchased cell lines were STR identified and compared to authoritative databases.
Mycoplasma contamination	All cell lines tested negative for mycoplasma contamination.
Commonly misidentified lines<br>(See ICLAC register)	There is no commonly misdentified cell lines used in the study .

Clinical data

Policy information about clinical studies

All manuscripts should comply with the ICMJE guidelines for publication of clinical research and a completed CONSORT checklist must be included with all submissions.

Clinical trial registration	The research was permitted by the Institutional Review Board of Tongji Hospital (Wuhan, China) (TJ-IRB20221137).
Study protocol	The full trial protocol can be accessed in the paper.
Data collection	From December 2020 to June 2022, a total of 109 IBC patients who have received neoadjuvant chemotherapy with doxorubicin were randomly admitted in the Tongji Hospital of Huazhong University of Science and Technology.
Outcomes	primary outcome :complete all chemotherapy regimens. secondary outcome :intolerance to chemotherapy or disease progression during chemotherapy
